# Cytotoxic and Antileishmanial Effects of the Monoterpene β-Ocimene

**DOI:** 10.3390/ph16020183

**Published:** 2023-01-26

**Authors:** Julyanne Maria Saraiva de Sousa, Thaís Amanda de Lima Nunes, Raiza Raianne Luz Rodrigues, João Paulo Araújo de Sousa, Maria da Conceição Albuquerque Val, Francisco Alex da Rocha Coelho, Airton Lucas Sousa dos Santos, Nicolle Barreira Maciel, Vanessa Maria Rodrigues de Souza, Yasmim Alves Aires Machado, Paulo Sérgio de Araújo Sousa, Alyne Rodrigues de Araújo, Jefferson Almeida Rocha, Damião Pergentino de Sousa, Marcos Vinicius da Silva, Daniel Dias Rufino Arcanjo, Klinger Antônio da Franca Rodrigues

**Affiliations:** 1 Laboratory of Infectious Diseases, Campus Ministro Reis Velloso, Parnaíba Delta Federal University, Parnaíba 64202-020, PI, Brazil; 2 Research Group on Medicinal Chemistry and Biotechnology, Federal University of Maranhão, São Bernardo 65550-000, MA, Brazil; 3 Research Center in Biodiversity and Biotechnology, Campus Ministro Reis Velloso, Parnaíba Delta Federal University, Parnaíba 64202-020, PI, Brazil; 4 Laboratory of Pharmaceutical Chemistry, Department of Pharmaceutical Sciences, Campus I, Federal University of Paraiba, João Pessoa 58051-900, PB, Brazil; 5 Laboratory of Immunology and Parasitology, Institute of Biological and Natural Sciences, Federal University of Triângulo Mineiro, Uberaba 38025-180, MG, Brazil; 6 Department of Biophysics and Physiology, Federal University of Piauí, Teresina 64049-550, PI, Brazil

**Keywords:** β-ocimene, antileishmanial, cytotoxicity, *L. amazonensis*, oxidative

## Abstract

Leishmaniasis is a group of infectious-parasitic diseases with high mortality rates, and endemic in many regions of the globe. The currently available drugs present serious problems such as high toxicity, costs, and the emergence of drug resistance. This has stimulated research into new antileishmania drugs based on natural products and their derivatives. β-Ocimene is a monoterpene found naturally in the essential oils of many plant species which presents antileishmanial activity, and which has not yet been evaluated for its potential to inhibit the etiological agent of leishmaniasis. The aim of this work was to evaluate the activity of β-ocimene against *Leishmania amazonensis*, its cytotoxicity, and potential mechanisms of action. β-Ocimene presented direct activity against the parasite, with excellent growth inhibition of promastigotes (IC_50_ = 2.78 μM) and axenic amastigotes (EC_50_ = 1.12 μM) at concentrations non-toxic to RAW 264.7 macrophages (CC_50_ = 114.5 µM). The effect is related to changes in membrane permeability and resulting abnormalities in the parasitic cell shape. These were, respectively, observed in membrane integrity and atomic force microscopy assays. β-Ocimene was also shown to act indirectly, with greater activity against intra-macrophagic amastigotes (EC_50_ = 0.89 μM), increasing TNF-α, nitric oxide (NO), and reactive oxygen species (ROS), with lysosomal effects, as well as promoting decreases in IL-10 and IL-6. Against intra-macrophagic amastigote forms the selectivity index was higher than the reference drugs, being 469.52 times more selective than meglumine antimoniate, and 42.88 times more selective than amphotericin B. Our results suggest that β-ocimene possesses promising in vitro antileishmania activity and is a potential candidate for investigation in in vivo assays.

## 1. Introduction

*Leishmania* spp. are protozoan parasites of the Trypanosomatidae family and the etiological agents of leishmaniasis. They are transmitted during blood meals taken by infected female phlebotomine sandflies [1]. According to the World Health Organization (WHO), leishmaniasis is also a neglected tropical disease (NTD), endemic in tropical and subtropical regions, with greater prevalence in developing countries. Leishmaniasis is considered a global public health problem, affecting approximately 1.3 million people per year, with 20,000 to 30,000 deaths [2].

Depending on the *Leishmania* spp. strain and host immunity, clinical manifestations differ significantly [3]. Two principal forms are reported: visceral leishmaniasis, which mainly affects internal organs such as the spleen, liver, and lymph nodes, and is the most severe clinical form; and cutaneous leishmaniasis, which causes ulcerated and non-ulcerated lesions in the skin and mucosal regions. Cutaneous leishmaniasis itself is subdivided into localized cutaneous leishmaniasis, disseminated cutaneous leishmaniasis, diffuse cutaneous leishmaniasis, and muco-cutaneous leishmaniasis (which can cause weakness, disfigurement, and even social withdrawal) [4].

Currently, there are no human vaccines, and leishmaniasis treatment is based on chemotherapy. Pentavalent antimonial drugs were introduced in the 1940s and are still the first-line treatment for leishmaniasis. They present many limitations such as high toxicity and cost, long treatment durations, and induction of resistance [5]. Second-line treatments include amphotericin B, miltefosine, paromomycin, and pentamidine, and these though effective, are even more toxic than pentavalent antimonials, which limits their use [6].

Due to these problems in leishmaniasis therapy, drugs with antileishmanial activity based on natural product derivatives are being sought. In this context, plant-derived volatile essential oils and their principal compounds (monoterpenes and sesquiterpenes) offer promising antileishmanial activity [7].

β-Ocimene (beta-3,7-dimethyl-1,3,6-octatriene) is an acyclic olefin monoterpene found in the essential oils of leaves and flowers of many plant species. It performs many biological functions, including use of its allopathic effect, in pesticides, and for defense against herbivores [8,9]. β-Ocimene also has great importance for agronomy, cosmetics, and the pharmaceutical industry. Noting its high amounts of methyl-SA and *cis*-3-hexen-1-ol (a C6 volatile), Perring et al. [10] treated tomato plants with β-ocimene and observed fewer parasites (such as aphids), β-ocimene is now a major component in cannabis essential oils as developed by an international medical cannabis producer [11]. β-ocimene is also approved by the Food and Drug Administration (FDA) as a food additive for human consumption, which makes its use as a medicine easier to justify.

β-ocimene is present in the compositions of many essential oils with proven antileishmanial activity such as *Pistacia lentiscus* L. [12] and *Piper marginatum* Jacq. [13]. β-ocimene is a major compound in *Syzygium cumini* (L.) Skeels. essential oil [14,15], yet despite being found in essential oils with proven antileishmanial action, its antileishmanial efficacy has not yet been evaluated. Thus, there is need to evaluate its activity in isolation as a new agent to treat leishmaniasis; the objective of this study was to evaluate the effects of β-ocimene on evolutionary forms of *Leishmania (Leishmania) amazonensis*, its cytotoxicity against mammalian cells, and its possible mechanisms of action.

## 2. Results

### 2.1. Antileishmanial Activity and Cytotoxic Effects of β-Ocimene

β-Ocimene presented cytotoxic effect against promastigote forms at all concentrations tested, with respective inhibition of 27.3%, 70.25%, 81.4%, and 100% at concentrations of 1.56 μM, 3.12 μM, 6.25 μM, and 12.5 μM, (Table 1), and with an inhibitory concentration for 50% of promastigotes (IC_50_) of 2.78 μM (Table 2). The cytotoxic profile of β-ocimene was evaluated against axenic amastigotes of *L. amazonensis.* After 72 h of exposure, the percentage of inhibition of the amastigote forms was, respectively, 59.06% and 79.11% at concentrations of 1.56 μM and 3.12 μM, and at concentrations of 6.25 μM and 12.5 μM, β-ocimene inhibited 100% of the amastigote forms (Table 1), with an EC_50_ of 1.12 μM (Table 2). For the reference drugs, the low activity of meglumine antimoniate was observed with an IC_50_ of 2150 μM for promastigotes and an EC_50_ of 1730 μM for axenic amastigotes (Table 2). Amphotericin B presented antileishmanial activity with an IC_50_ of 0.35 μM and an EC_50_ of 0.51 μM (respectively) for promastigotes and axenic amastigotes (Table 2).

β-Ocimene presented cytotoxicity against RAW 264.7 macrophages and Vero cells, yet only at high concentrations, as shown in Table 1. β-Ocimene significantly reduced macrophage viability at concentrations of 100 μM (46.3%), 200 μM (56.58%), and 400 μM (81.7%), and Vero cells at concentrations of 200 μM (36.2%) and 400 μM (69.18%) (Table 1). The cytotoxic concentration for 50% of RAW 264.7 macrophages (CC_50_) presented by β-ocimene was, respectively, 114.5 µM for RAW 264.7 macrophages, and 298.54 µM for Vero cells (Table 2).

According to the SI (Table 2), β-ocimene was more toxic to the different parasite forms (promastigote, axenic amastigote, and intra-macrophagic amastigote) than to macrophages, indicating a good SI. In addition, the compound in question presented a higher selectivity index than the reference drugs, being 469.52 times more selective than meglumine antimoniate, and 42.88 times more selective than amphotericin B, as compared to the intra-macrophagic amastigote form.

### 2.2. Membrane Integrity Assay

**Figure 1:**
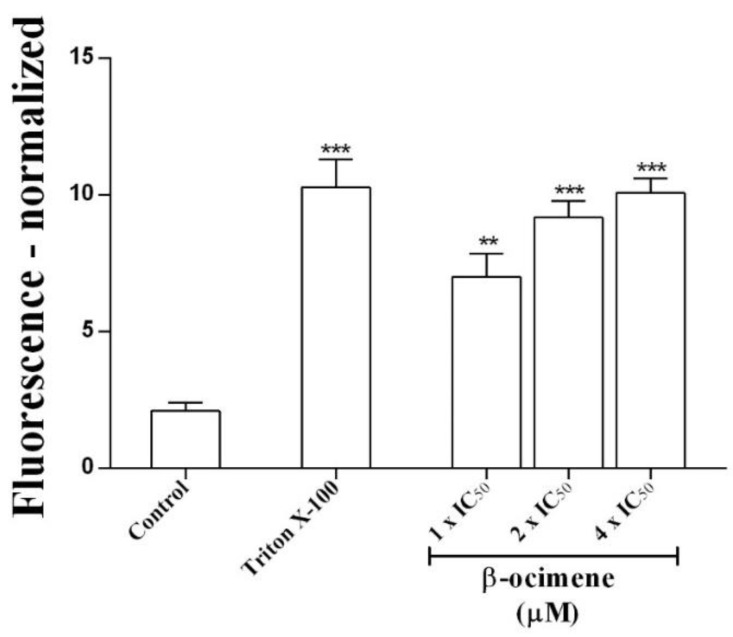
Effects of β-ocimene on *Leishmania amazonensis* promastigote plasmatic membrane permeability. Cells (10^6^/well) were incubated at 26 °C for 7 h in the absence or presence of β-ocimene at IC_50_, 2 × IC_50_, and at 4 × IC_50_. A SYTOX green probe was used. Data represent the normalized fluorescent mean ± standard error for three independent experiments carried out in triplicate. (**) *p <* 0.01 vs. control; (***) *p <* 0.001 vs. control.

To determine possible damage to the parasite membrane, membrane permeability assays were performed. A significant increase in permeability was observed (via fluorescent signal) in promastigotes treated with β-ocimene at IC_50_ (*p* < 0.01), 2 × IC_50_ (*p* < 0.001), and at 4 × IC_50_ (*p* < 0.001), similar to the positive control—promastigotes were treated with 0.1% Triton X-100 (Figure 1).

### 2.3. Morphological Alterations in β-Ocimene Treated Promastigote Forms

**Figure 2:**
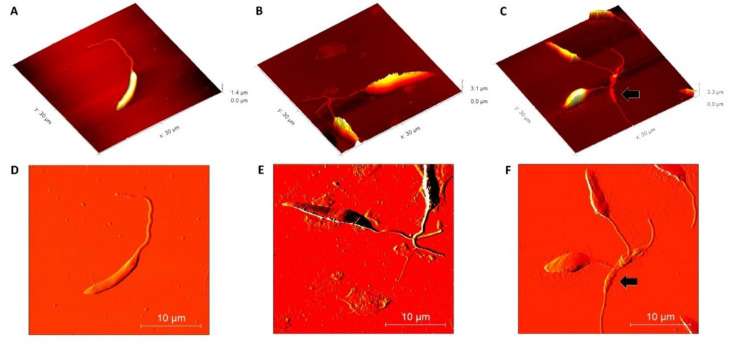
Morphological analysis of *Leishmania amazonensis* using atomic force microscopy (above: 2D amplitude images; below: 3D height images). (**A**,**D**): control (untreated); (**B**,**E**): β-ocimene treated (IC_50_); (**C**,**F**): treated with β-ocimene (2 × IC_50_). Black arrow: ultrastructural alteration.

Once the effective action of β-ocimene on *L. amazonensis* was observed, morphological alterations were evaluated using AFM techniques. Using the AFM deflection image, it was observed that the untreated promastigotes presented normal membrane texture and were topographically vacuolated. The presence of a long, exteriorized flagellum confirmed cell normality (Figure 2A,D). However, as compared to the controls (untreated), at concentrations of 1 × IC_50_ (Figure 2B,E) and 2 × IC_50_ (Figure 2C,F), the cell surface ultra-structures were topographically altered by the β-ocimene treatment. Loose membrane texture and abnormalities in the cell shape were also observed upon treatment with β-ocimene at concentrations of 1 × IC_50_ and 2 × IC_50_.

### 2.4. In Vitro Efficacy of β-Ocimene against Intra-Macrophage Amastigote Forms

**Figure 3:**
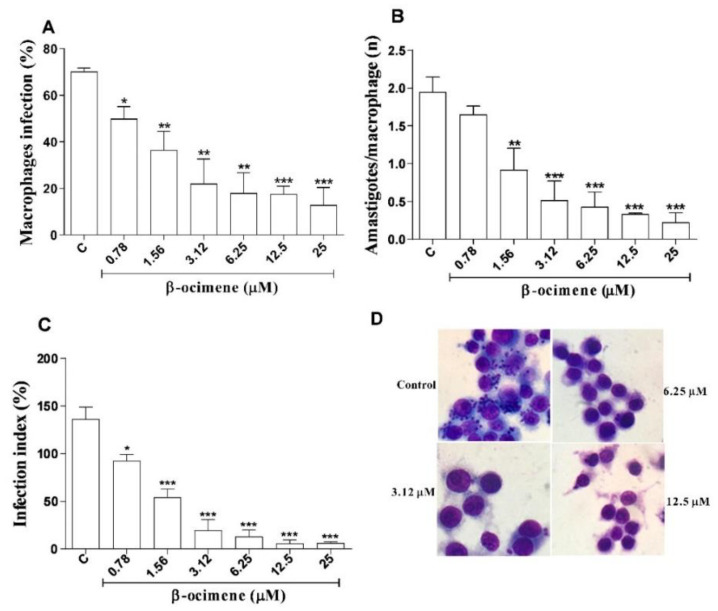
Effect of β-ocimene in the treatment of RAW 264.7 macrophages infected with *Leishmania amazonensis* for 72 h. (**A**) Percentage of infected macrophages; (**B**) number of amastigotes per infected macrophage; (**C**) infection index, and (**D**) digital optical microscopy photograph (1000× magnification) of a representative experiment after 72 h of treatment. Macrophages RAW 264.7 attached to 13 mm coverslips were infected with 10^6^ *Leishmania amazonensis* promastigotes for each macrophage. Afterwards, they were treated with different concentration of β-ocimene at 37 °C and 5% CO_2_ for 72 h. After this treatment, the coverslips were stained and analyzed using light microscopy. The graph represents the mean ± standard error of the mean of three independent experiments performed in triplicate. One-way ANOVA followed by Tukey’s post-test was performed for comparison between groups with (*) *p* < 0.05; (**) *p* < 0.01 and (***) *p* < 0.001 compared to control. C—Control (0.5% DMSO in complete DMEM).

The results for β-ocimene treatment of macrophages infected with *L. amazonensis* are shown in Figure 3, using three criteria: infection percentage, number of amastigotes per macrophage, and infection index. At low concentrations, β-ocimene was able to reduce the percentage of infected macrophages (Figure 3A,D), the number of amastigotes per macrophage (Figure 3B), and also the infection rate (Figure 3C). β-Ocimene generated an effective concentration against 50% of amastigotes (EC_50_) of 0.89 μM, while meglumine antimoniate and amphotericin B, respectively, generated EC_50_ values of 577.5 and 0.13 μM (Table 2).

### 2.5. Immunomodulatory Activity

Upon observing a reduction in the rate of macrophage infection, and better results for the intra-macrophagic amastigote form than for promastigotes and axenic amastigotes, we evaluated the hypothesis that the antileishmania activity could be correlated with immunomodulatory activity.

**Figure 4:**
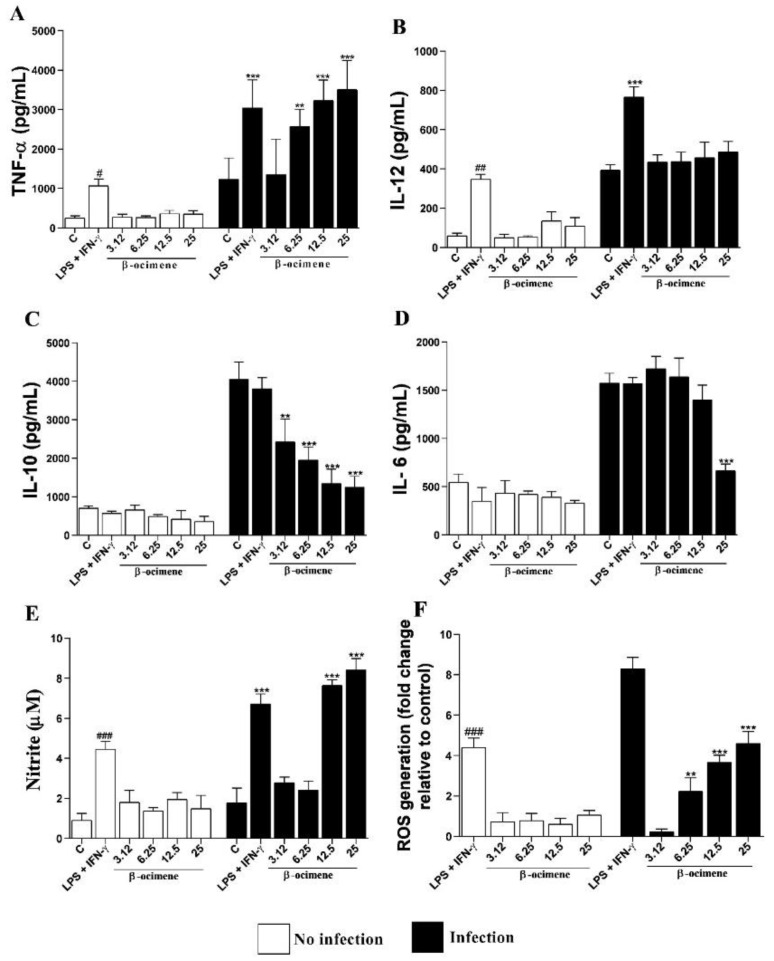
Levels of TNF-α (**A**), IL-12 (**B**), IL-10 (**C**), IL-6 (**D**) cytokines, and NO (**E**) and ROS (**F**) produced by RAW 264.7 macrophages uninfected and infected with *Leishmania amazonensis* and treated with β-ocimene for 72 h at 37 °C and 5% CO_2_. The results represent means ± standard error for three independent experiments carried out in triplicate. (#) *p* < 0.05 vs. uninfected control; (##) *p* < 0.01 vs. uninfected control; (###) *p* < 0.001 vs. uninfected control; (**) *p* < 0.01 vs. infected control; (***) *p* < 0.001 vs. infected control. C—control. LPS—lipopolysaccharide.

As shown in Figure 4, no significant differences were observed in the cytokine levels of uninfected macrophages and treated with β-ocimene, compared to the control. However, β-ocimene induced a host-protective cytokine response, an increase in the production of TNF-α (Figure 4A), NO (Figure 4E), and ROS (Figure 4F), with a decrease in levels of IL-10 (Figure 4C) and IL-6 (Figure 4D) in RAW 264.7 macrophages infected with *L. amazonensis*. Β-Ocimene did not affect IL-12 levels under the evaluated conditions (Figure 4B).

**Figure 5:**
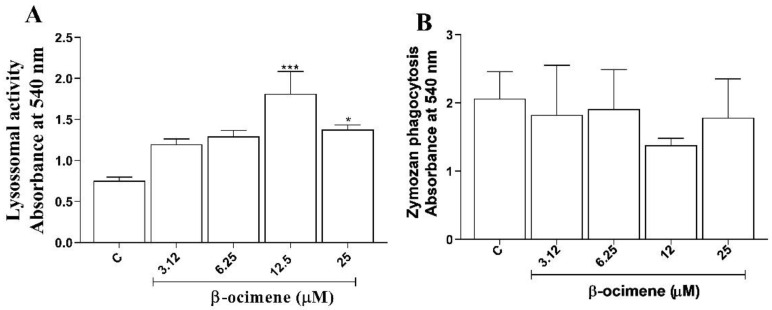
The influence of β-ocimene on the lysosomal volume (**A**) and phagocytic activity (**B**). RAW 267.4 macrophages were treated at a range of concentrations for 72 h. Lysosomal activity was analyzed spectrophotometrically by quantifying the increase in neutral red (NR) uptake following solubilization with the extraction solution. The phagocytosis was analyzed by incorporation of NR-stained zymosan, solubilized with the extraction solution. Data are presented as mean ± S.E.M. of three experiments carried out in triplicate. (*) *p* < 0.05 vs. control; (***) *p* < 0.001 vs. control. C—control.

Macrophage activation parameters were evaluated based on the retention of neutral red and zymosan by macrophages treated with β-ocimene yet not stimulated by *L. amazonensis.* As shown in Figure 5A, β-ocimene promoted an increase in the volume of lysosomal vesicles at concentrations of 12.5 and 25 µM. On the other hand, no increase in phagocytosis of zymosan particles was observed with β-ocimene treatment under the test conditions (Figure 5B).

## 3. Discussion

The antileishmanial activity of β-ocimene was initially evaluated against *L. amazonensis* promastigotes and axenic amastigotes to observe direct effects and the ability of the compound to inhibit the growth of the parasite in vitro. The compound was more effective in inhibiting the growth of *Leishmania* sp. promastigote forms with better IC_50_ values than other monoterpenes considered promising. The compound was more effective in inhibiting the growth of *Leishmania* sp. promastigote forms with better IC_50_ values than other monoterpenes considered promising. For example, in a study by Youssefi et al. [16], the antileishmanial activity of the monoterpenes thymol and carvacrol against *L. infantum* promastigotes were evaluated. Both presented activities, with respective IC_50_ values of 48 µM and 65 µM. Another monoterpene considered promising, limonene, presented activity against *L. amazonensis* promastigotes with an IC_50_ value of 252 μM [17]. As with the promastigote form, β-ocimene presents direct activity against axenic amastigote forms, with an even more expressive inhibitory effect. In previous works with *L. amazonensis*, the antileishmania activity of 4-nitrobenzaldehyde thiosemicarbazone alone (IC_50_ value of 8 μM), an (*S*)-limonene derivative, has been reported against axenic amastigotes [18].

In order to obtain a substance with selective antileishmanial activity for the parasite and with low toxicity to cells, a cytotoxicity assay was performed in RAW 264.7 macrophages and Vero cells. When the SI was calculated, the compound was 41.18 times more toxic to *L. amazonensis* promastigotes, 102.23 times more toxic to axenic amastigotes, and 128.65 times more toxic to intra-macrophage amastigotes than to macrophages. The selectivity index assesses how much more selective a compound is towards the parasite than to the cell. Substances presenting an SI greater than 20 are considered safe [19]. β-Ocimene was more effective and safer in macrophage-internalized amastigotes. This efficacy may be associated with mechanisms of macrophage activation and induction of immune response by the cell [20]. The reference drugs tested in this study presented low SIs, which corroborates with the known high toxicity and side effects that these drugs possess [6].

Considering the antileishmania effectiveness of β-ocimene, its mechanisms of action against the parasite were evaluated. Membrane integrity assays, and immunomodulatory activity studies (in vitro) were performed.

It was demonstrated that β-ocimene acts directly on the parasite. We found that β-ocimene alters plasma membrane permeability, as observed in an experiment using SYTOX green, a high-affinity nucleic acid stain that penetrates cells with compromised plasma membranes and (upon nucleic acid binding) augments their fluorescence by more than 500-fold [21]. The alteration of plasma membrane permeability observed for β-ocimene corroborates many studies, demonstrating that essential oils and their isolated constituents are able to interact with plasma membrane molecules, such as proteins and lipids, and promote decreased rigidity or even leakage of intracellular parasite content [15]. Further, essential oils are already known to interact with internal cell targets, disrupting important metabolic pathways, and decreasing mitochondrial membrane potential to promote both cell necrosis and apoptosis [14].

In addition to membrane permeability, structural changes caused by β-ocimene in parasites at the nanometer scale were evaluated through atomic force microscopy (MFA). After treatment with β-ocimene, changes were observed in both the membranes and shapes of the parasites. Cell retraction, membrane alteration, and vesicle formation are typical characteristics of cells undergoing apoptosis [22]. Such characteristics evidenced in this study indicate that β-ocimene acts on the parasite through induction of apoptosis.

In response to *Leishmania* spp. infection, macrophages commence phagocytosis, and production of cytokines and microbicidal molecules (such as reactive oxygen and NO species) [20]. Our study evaluated the activation mechanism through structural alterations (lysosomal volume increases and phagocytosis) and through cellular mechanisms (TNF-α, IL-6, IL-10, IL-12, ROS, and NO production).

Viable and metabolically active cells are able to take up neutral red dye through lysosomal vesicles [23]. In this study, the ability of β-ocimene to increase retention of neutral red by macrophages was analyzed. It was shown that macrophages treated with β-ocimene presented modulated endocytic compartment volumes, suggestive of an increase in the cell’s defense potential.

In macrophages not stimulated with *L. amazonensis,* phagocytic capacity was analyzed using zymosan particle incorporation. Zymosan particles are readily phagocytized by viable macrophages and present a proven ability to stimulate a Th1-type response [24]. In this study, the treatment of macrophages with β-ocimene did not increase phagocytic capacity, suggesting that the pathway is not involved in macrophage activation.

Investigating cellular mechanisms, our study demonstrated that β-ocimene (at the highest concentrations tested on macrophages stimulated by *L. amazonensis*) increased production of Th1 cytokines (TNF-α), ROS, and NO, and reduced levels of the Th2 profile cytokines (IL-6 and IL-10). Previous studies have shown that increased production of Th1 cytokines and reduction of Th2 cytokines is associable with parasite control and is a target for new antileishmanial drugs [25].

There are two macrophage activation signals in the innate immune response to infection by *Leishmania* sp.: first, NK cells (natural killers) produce INF-γ; and secondly, activation of Toll-like receptors (TLRs) induces production of TNF-α (associated with the activation of macrophages to fight the parasite) [26]. INF-γ induces differentiation of Th0 into Th1, and upon expression of Th1 cytokines (TNF-α, IL-12, and INF-γ), activation of the inducible nitric oxide synthase enzyme (iNOS) occurs with consequent production of NO (a microbicidal molecule that induces parasite death through oxidative stress) [27]. In this study it was evidenced that in macrophages stimulated by *L. amazonensis,* β-ocimene (at the highest concentrations tested) induced production of ROS and NO. Yet according to Vouldoukis et al. [28], an increase in cytokine IL-10 is associated with a Th2 response to iNOS suppression, and consequently a reduction in NO production, inhibiting macrophage activation and thus favoring parasite proliferation.

## 4. Materials and Methods

### 4.1. Chemicals and Pharmaceuticals

**Figure 6:**
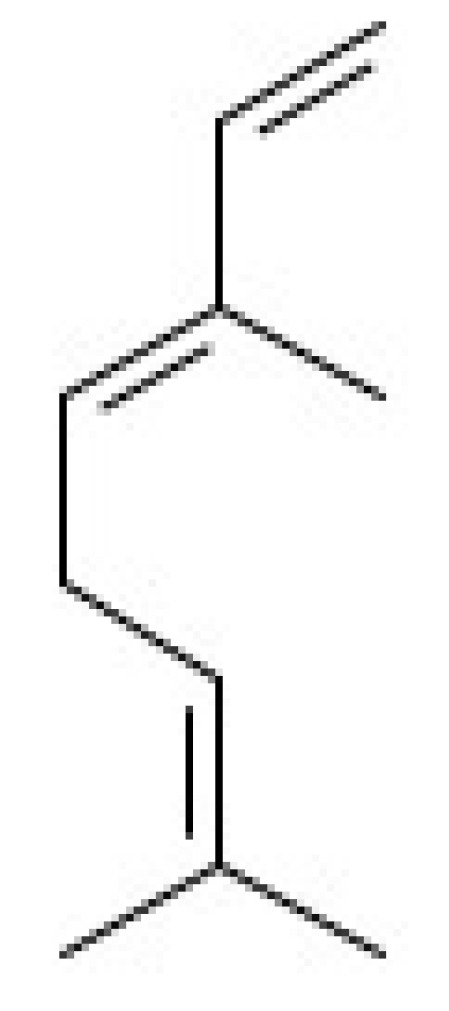
Chemical structure of β-ocimene.

Cell culture medium (DMEM and Schneider), antibiotic solution (penicillin and streptomycin), 3-(4,5-100 dimethylthiazol-2-yl) 2,5-bromide diphenyltetrazolium (MTT), zymosan particles, Griess reagent (1% sulfanilamide in 10% (*v*/*v*) H_3_PO_4_ in Milli-Q water), and neutral red, were purchased from Merck Company (São Paulo, SP, Brazil). Fetal bovine serum (FBS) was obtained from ThermoFisher (São Paulo, SP, Brazil). Interferon gamma (IFN-γ) and lipopolysaccharide (LPS) were obtained from Life Technologies Corporation (Frederick MD, USA). (*E*)-β-ocimene (C_10_H_16_; ≥90% purity; its structure is shown in Figure 6; simply called β-ocimene in this work) was obtained from LGC Standards (London, UK). The murine standard TMB ELISA Development Kit was obtained from eBioscience (São Paulo, SP, Brazil). Sodium dodecyl sulfate (SDS) and dimethyl sulfoxide (99% DMSO) were obtained from Mallinckrodt Chemicals (St. Louis, MO, USA). Sodium nitrite (NaNO_2_) was obtained from Vertec Fine Chemistry (Rio de Janeiro, RJ, Brazil).

### 4.2. Drug Preparation

For all in vitro tests, stock solutions of β-ocimene and reference drugs were prepared using DMSO as a vehicle at a concentration of 80 mg/mL. For each test, the appropriate stock solution was chosen and diluted in the culture media until the desired final concentration was reached.

### 4.3. Maintenance of the Parasites and Macrophages

Strains of *Leishmania (Leishmania) amazonensis* (IFLA/BR/67/PH8) (promastigote form) were cultivated in Schneider’s insect medium (supplemented with 10% FBS, and 1% antibiotic solution), at 26 °C, and at pH 7, in a biological oxygen demand (BOD) incubator. To transform the promastigote forms into axenic amastigotes, the temperature was increased to 32 °C and the pH of the medium was adjusted to 5.5 [14].

RAW 264.7 lineage macrophages (available from the cell bank in Rio de Janeiro, Brazil) were used, grown in sterile bottles with DMEM medium, pH 7.2 containing 10% FBS and 1% penicillin 100 U/mL and streptomycin 100 μg/mL, and kept in an oven at 37 °C and 5% CO_2_.

### 4.4. In Vitro Inhibition Assay of β-Ocimene on Promastigotes and Axenic Amastigotes

Promastigote and axenic amastigote growth inhibition was tested as previously described by DIAS et al. [29]. In a 96-well plate, parasites were seeded at 10^6^ parasites per well in 100 μL of supplemented Schneider medium. The parasites were treated with differing concentrations of β-ocimene (1.56–12.5 μM), and reference drugs, meglumine antimoniate (500–40,000 μM) and amphotericin B (0.062–0.5 μM). The plates were incubated in a BOD incubator at 26 °C for 72 h. Afterwards, 10 µL of MTT (5 mg/mL) was added to each well, and the plate was incubated again for 4 h, when 50 µL of 10% SDS was added. Absorbances were then measured using an ELISA plate reader (BioSystems model ELx800, Curitiba, PR, Brazil) at 540 nm. The negative control consisted of Schneider medium with 0.5% DMSO and was considered as 0% inhibition.

### 4.5. Membrane Integrity Assay

Plasma membrane integrity in promastigote forms of *L. amazonensis* treated with β-ocimene was evaluated using the SYTOX green dye method [30]. Promastigote forms in logarithmic growth were seeded in Schneider medium and exposed to β-ocimene at concentrations corresponding to IC_50_, 2 × IC_50_ and 4 × IC_50_ in the presence of SYTOX green dye (5 μM) for 7 h at 26 °C in a BOD incubator. The plates were then read in a fluorescence reader with an emission filter of 523 nm and excitation at 488 nm.

### 4.6. Parasite Morphological Evaluation Using Atomic Force Microscopy (AFM)

Promastigote forms of *L. amazonensis* were grown in 96-well plates at a concentration of 10^6^ per well in complete Schneider medium. β-ocimene was added at concentrations equivalent to IC_50,_ 2 × IC_50_, and 4 × IC_50_, and incubated at 26 °C for 72 h in BOD, and the parasites were then washed twice with ice-cold PBS (under 1100× *g* for 15 min at room temperature), and then fixed with 2.5% (*v*/*v*) glutaraldehyde in PBS, pH 7.2, for 1 h. Morphological analysis using AFM (TT-AFM equipment, AFM Workshop-USA), at a resonant frequency of approximately 242 kHz, was then performed [31].

### 4.7. Cytotoxic Effect on Mammalian Cells and the Calculation of Selective Index (SI)

In a 96-well plate, RAW 264.7 macrophages were plated at 10^5^ cells per well in 100 μL of supplemented DMEM medium. The plate was incubated for 3 h at 37 °C and 5% CO_2_ for cell adhesion. Afterwards, to remove non-adhered cells, washing with PBS (phosphate-buffered saline solution) was performed. Furthermore, 100 μL of DMEM supplemented with β-ocimene (3.12–400 μM), and the reference drugs, meglumine antimoniate (250–2000 μM) and amphotericin B (0.062–0.5 μM) were then added to the wells, with oven incubation at 37 °C, and 5% CO_2_ for 72 h. Then, 10 μL of MTT (5 mg/mL) per well was then added, being kept for another 4 h in an oven at 37 °C and 5% CO_2_. The supernatant was removed, and 100 µL of DMSO was added to all wells. After 30 min of stirring, readings were taken at 540 nm. DMEM supplemented with 0.5% DMSO was used as a negative control and considered as 100% macrophage viability. The selectivity index for each treatment was calculated by dividing the CC_50_ by the IC_50_, or EC_50_ [32].

### 4.8. Treatment of Infected Macrophages

In a 24-well plate with 13 mm round sterile cover slips, RAW 264.7 macrophages were added (at a concentration of 10^5^ cells to 1 mL of supplemented DMEM. For cell adhesion, the plates were incubated at 37 °C and 5% CO_2_ for 3 h. Three washes were then performed with PBS at 37 °C to discard non-adherent cells, after which 1 mL of DMEM supplemented (stationary phase) promastigote forms of *L. amazonensis* was added at the ratio of 10 promastigotes per macrophage. The plate was then incubated at 5% CO_2_ and 37 °C for 4 h. After infection, the medium was aspirated and the treatment was added using 1 mL of DMEM supplemented with β-ocimene (3.12–25 μM) with the reference drugs, meglumine antimoniate (1.56–200 μM) and amphotericin B (0.031–2 μM), this was subsequently incubated at 37 °C and 5% CO_2_ for 72 h. Finally, the coverslips were removed, fixed, stained, and mounted on permanent slides using Entellan. The number of infected macrophages and the number of parasites per macrophage were counted: 300 cells were evaluated on each slide at 1000× magnification. The infection index was calculated by multiplying the number of infected macrophages by the number of amastigotes per infected macrophage. The negative control was performed using DMEM with 0.5% DMSO. The supernatant was removed at the end of the experiment and kept at −20 °C for later use in nitric oxide dosing tests [30].

### 4.9. Lysosomal Activity

RAW 264.7 macrophages were added (10^5^ cells per well) to the 96-well plate in 100 μL of DMEM with different concentrations of β-ocimene (3.12–25 μM). The plate was incubated for 72 h at 37 °C and 5% CO_2_. Afterwards, 10 μL of neutral red 0.2% DMSO solution was added and incubated for 30 min. The supernatant was then removed, and the wells were washed with 0.9% saline at 37 °C, and 100 μL of extraction solution was added to dissolve the neutral red inside the lysosomal secretion vesicles. After 30 min on a Kline shaker, the plate was read at 540 nm [33].

### 4.10. Phagocytosis Assay

RAW 264.7 macrophages were plated in a 96-well plate at 10^5^ cells per well in 100 μL of DMEM medium containing different concentrations of β-ocimene (3.12–25 μM). The plate was incubated for 72 h at 37 °C and 5% CO_2_; after which 10 μL of stained zymosan per well was added, and the plate conditioned for 30 min at 37 °C. Then, 100 μL of Baker’s fixative (4% *v*/*v* formaldehyde, 2% *w*/*v* sodium chloride, and 1% *w*/*v* calcium acetate in distilled water) was added to stop phagocytosis, and 30 min later, the plate was washed with 0.9% saline to remove the zymosan not phagocytized by macrophages. Afterwards, the supernatant was removed and 100 μL of extraction solution was added and solubilized in a Kline shaker. Absorbances were measured at 540 nm [33].

### 4.11. Estimation of Th1/Th2 Cytokine and Nitric Oxide (NO) Production

Modulation of Th1 (TNF-α, IL-12), Th2 (IL-10, IL-6), and NO cytokine production was investigated in the supernatants of infected macrophages, whether treated or untreated with β-ocimene. Cytokine concentration (pg/mL) in macrophage supernatants was determined using the ELISA sandwich technique according to the manufacturer’s (eBioscience) instructions, using interpolation with a standard curve of recombinant cytokines. Optical density was read at 450 nm using a microplate spectrophotometer.

Nitrite levels from the supernatants of the infected macrophages, whether treated or untreated with β-ocimene, were used indirectly to estimate NO production. The NO concentration (µM) was determined using the Griess reaction following a previously described methodology [32], by interpolation with a standard curve with NaNO_2_.

LPS (1 μg/mL) plus IFN-γ (1000 U/mL) were used as a positive control.

### 4.12. Measurement of Reactive Oxygen Species (ROS)

ROS levels, as produced by the macrophages infected with *L. amazonensis* and treated with β-ocimene, were determined by the H_2_DCFDA test [34]. Macrophages were infected according to Section 2.5 and treated with β-ocimene (3.12–12.6 µM) for 72 h. At the end of the period, 10 µL of 20 µM H_2_ DCFDA dye was added. The plate was incubated at 37 °C for 30 min (in the dark), and the fluorescence intensity was measured using a spectrofluorometer (FLx800) with excitation at 485 nm and emission at 528 nm.

### 4.13. Statistical Analysis

To determine the CI_50_, EC_50_, and CC_50_ with a confidence limit of 95%, the probit regression model from SPSS 13.0 was used. For comparisons between groups, ANOVA analysis of variance was performed followed by Tukey’s test using GraphPad Prism version 8.0, and taking the value of *p* < 0.05 as the maximum level of statistical significance.

## 5. Conclusions

In conclusion, our results reveal that β-ocimene exhibits selective antileishmanial activity against both stages of *L. amazonensis* with low levels of cytotoxicity to host cells. The effects of β-ocimene against the parasite are associated with compromised plasma membrane integrity. Our study also revealed that the antileishmanial activity of β-ocimene involves indirect immunomodulation; evidenced by increases in lysosomal activity and levels of TNF-α, NO, and ROS, and decreases in IL-10 and IL-6. These results encourage further studies with this compound in in vivo models of tegumentary leishmaniasis and synergistic studies with reference drugs, towards development of new antileishmania agents.

## Figures and Tables

**Table 1:** Cytotoxic effects of β-ocimene on *Leishmania (Leishmania) amazonensis* promastigotes and axenic amastigotes, and RAW 264.7 macrophages. Data represent the average cytotoxicity percentage (%) ± standard error for three independent experiments performed in duplicate, considering the control group as 0% cytotoxicity; (^a^) *p* < 0.05 vs. control; (^b^) *p* < 0.01 vs. control; (^c^) *p* < 0.001 vs. control.

Concentrations	% Cytotoxicity			
	Promastigotes	Axenic Amastigotes	RAW 264.7 Macrophages	Vero Cells
1.56 µM	27.3 ± 2.16 ^b^	59.06 ± 2.4 ^c^	-	-
3.12 µM	70.25 ± 2.2 ^c^	79.11 ± 3.7 ^c^	-	-
6.25 µM	81.4 ^c^	100 ^c^	-	-
12.5 µM	100 ^c^	100 ^c^	-	-
50 µM	-	-	1.17 ± 0.09	1.14 ± 0.1
100 µM	-	-	46.3 ± 4.11 ^b^	6.04 ± 0.41
200 µM	-	-	56.58 ± 5.92 ^c^	36.2 ± 4.26 ^a^
400 µM	-	-	81.7 ± 8.16 ^c^	69.18 ± 4.9 ^c^

**Table 2:** Effect of β-ocimene and reference drugs on RAW 264.7 macrophage and Vero cell viability, antileishmanial activity, and selectivity index (SI). Data represent the average ± standard error for three independent experiments performed in triplicate. The differing mean concentrations against promastigote (IC_50_), axenic amastigote, and intra-macrophagic amastigote (EC_50_), macrophages, and Vero cells (CC_50_) were calculated using non-linear regression. SI (selectivity index) = CC_50 Raw 264.7_/IC_50_ or EC_50_.

Compounds	RAW 264.7	VERO Cells	Promastigotes		Axenic Amastigotes		Intramacrophagic Amastigotes	
	CC_50_ µM	CC_50_ µM	IC_50_ µM	SI	EC_50_ µM	SI	EC_50_ µM	SI
**β-ocimene**	114.5 *±* 8.2	298.54 *±* 7.17	2.78 *±* 0.17	41.18	1.12 *±* 0.05	102.23	0.89 *±* 0.03	128.65
**Amphotericin B**	0.39 *±* 0.03	2.46 *±* 0.6	0.35 *±* 0.09	1.11	0.51 *±* 0.02	0.76	0.13 *±* 0.4	3
**Meglumine** **antimoniate**	15,863 *±* 95.71	22,831 *±* 103.61	2150 *±* 31	0.73	1730 *±* 36.8	9.16	577.5 *±* 19.1	0.274

## Data Availability

Data is contained within the article.
